# Comparison of the protection of ferrets against pandemic 2009 influenza A virus (H1N1) by 244 DI influenza virus and oseltamivir

**DOI:** 10.1016/j.antiviral.2012.09.017

**Published:** 2012-12

**Authors:** Nigel J. Dimmock, Brian K. Dove, Bo Meng, Paul D. Scott, Irene Taylor, Linda Cheung, Bassam Hallis, Anthony C. Marriott, Miles W. Carroll, Andrew J. Easton

**Affiliations:** a School of Life Sciences, University of Warwick, Coventry CV4 7AL, UK; b Health Protection Agency, Porton Down, Salisbury SP4 0JG, UK

**Keywords:** Influenza virus, Defective interfering virus, Ferret, Protection, Oseltamivir

## Abstract

The main antivirals employed to combat seasonal and pandemic influenza are oseltamivir and zanamivir which act by inhibiting the virus-encoded neuraminidase. These have to be deployed close to the time of infection and antiviral resistance to the more widely used oseltamivir has arisen relatively rapidly. Defective interfering (DI) influenza virus is a natural antiviral that works in a different way to oseltamivir and zanamivir, and a cloned version (segment 1 244 DI RNA in a cloned A/PR/8/34 virus; 244/PR8) has proved effective in preclinical studies in mice. The active principle is the DI RNA, and this is thought to interact with all influenza A viruses by inhibiting RNA virus synthesis and packaging of the cognate virion RNA into nascent DI virus particles. We have compared the ability of DI virus and oseltamivir to protect ferrets from intranasal 2009 pandemic influenza virus A/California/04/09 (A/Cal, H1N1). Ferrets were treated with a single 2 μg intranasal dose of 244 DI RNA delivered as 244/PR8 virus, or a total of 25 mg/kg body weight of oseltamivir given as 10 oral doses over 5 days. Both DI virus and oseltamivir reduced day 2 infectivity and the influx of cells into nasal fluids, and permitted the development of adaptive immunity. However DI virus, but not oseltamivir, significantly reduced weight loss, facilitated better weight gain, reduced respiratory disease, and reduced infectivity on days 4 and 6. 244 DI RNA was amplified by A/Cal by >25,000-fold, consistent with the amelioration of clinical disease. Treatment with DI virus did not delay clearance or cause persistence of infectious virus or DI RNA. Thus in this system DI virus was overall more effective than oseltamivir in combatting pandemic A/California/04/09.

## Highlights

► We compared the ability of DI RNA and Tamiflu to protect ferrets from influenza. ► We treated ferrets with one 2 μg intranasal dose of DI RNA delivered as DI virus. ► Or we treated ferrets with 10 oral doses of Tamiflu totalling 25 mg over 5 days. ► Pandemic A/California/04/09 amplified the DI RNA by >25,000-fold. ► DI virus was more effective than Tamiflu in combatting pandemic A/California/04/09.

## 1 Introduction

Defective interfering (DI) viruses are natural mutants that arise spontaneously, occur widely, and have a genome that has undergone at least one major deletion. As a result their replication is dependent on complementation by a genetically compatible infectious helper virus to provide any missing function. All DI genomes retain sequences that allow them to be packaged and replicated. The resulting DI virus particle is usually indistinguishable from that of the infectious virus. In cell culture DI viruses are not only defective but also interfering, the DI genome being the structure responsible for this property. Thus, under appropriate conditions, the presence of the DI genome reduces the amount of infectious progeny virus produced (Holland, 1990a,b; Huang and Baltimore, 1970; Nayak et al., 1989; Perrault, 1981). Some, but not all DI viruses can protect animals from clinical disease caused by the homologous virus (Barrett and Dimmock, 1986; Dimmock, 1991, 1996; Dimmock et al., 2008; Roux et al., 1991). Influenza DI 244 virus also protects against genetically unrelated (heterologous) viruses (pneumonia virus of mice (PVM: *Paramyxoviridae* and influenza B virus) *in vivo*, primarily by induction of type 1 interferon (Easton et al., 2011; Scott et al., 2011b). DI virus-induced interferon is not required for protection against a lethal challenge with influenza A viruses (Easton et al., 2011).

Influenza A DI viruses were the first to be described (von Magnus, 1954) and have been studied extensively (Nayak, 1980; Nayak et al., 1985, 1989). However, most DI influenza virus preparations contain many different defective RNA sequences, so that it was not possible to determine the relationship between a particular defective RNA sequence and its biological properties. Recently we solved this problem using reverse genetics to make cloned DI viruses that contain one major species of DI RNA (Dimmock et al., 2008; Duhaut and Dimmock, 1998, 2000, 2002, 2003). One such influenza virus, containing the 244 DI RNA, derived from segment 1, strongly protected mice against disease caused by several different influenza A virus subtypes when inoculated intranasally (Dimmock et al., 2008). This protection is dependent on the integrity of the 244 DI RNA and protection is lost when the DI RNA is destroyed by extensive UV irradiation. The DI influenza A virus particle retains receptor specificity and, when topically applied, targets the DI RNA to influenza virus-susceptible cells in the respiratory tract. In this way the virus particle provides a highly specific delivery system.

We have shown that non-cloned and cloned DI influenza A viruses protect mice from lethal infection caused by influenza A virus (Dimmock et al., 1986, 2008; Morgan et al., 1993; Noble and Dimmock, 1994). Only a single dose of cloned 244/PR8 virus is needed and the DI RNA is amplified by the infecting virus (Scott et al., 2011a). Protection lasts for one to several weeks depending on the initial dose of DI virus (Dimmock et al., 2008; Scott et al., 2011a). Post-infection treatment is effective for up to 2 days after infection (Dimmock et al., 2008).

Influenza viruses usually have to be adapted to grow in mice whereas the ferret is highly susceptible to new human isolates (Francis, 1934; Shope, 1931; Smith et al., 1933). Because humans and ferrets develop a very similar disease the ferret is the preferred model for human influenza (Barnard, 2009; Cameron et al., 2008; Herlocher et al., 2001; Matsuoka et al., 2009; Smith and Sweet, 1988; van den Brand et al., 2010; Whitley, 2010). In a preliminary study in ferrets we tested a non-cloned DI influenza virus preparation that contained an unspecified collection of DI RNAs derived from an equine influenza A virus, and showed that it afforded ferrets some protection against an H3N2 challenge virus (Mann et al., 2006). More recently we used a cloned 244/PR8 virus (Dimmock et al., 2008) reconstructed with a PR8 hemagglutinin variant that bound to both α 2,6- and α 2,3-linked sialyl receptors (Meng et al., 2010), so that DI RNA would be delivered to and protect cells bearing both types of receptor. There are a number of studies of pandemic H1N1 in ferrets (Itoh et al., 2009; Maines et al., 2009; Munster et al., 2009; van den Brand et al., 2010). Pandemic 2009 H1N1 viruses differ from seasonal H1N1 viruses in a number of ways: the former use 2,6- and 2,3-linked receptors (Childs et al., 2009), preferentially infect the lower respiratory tract in humans and in animal models (Guarner and Falcon-Escobedo, 2009), replicate in the lower respiratory tract of ferrets rather than the nasal cavity (Munster et al., 2009; van den Brand et al., 2010), and cause more severe pathological lesions than seasonal H1N1 virus in mice, ferrets and non-human primates (Itoh et al., 2009).

In addition to vaccines (Cox and Bridges, 2007; Nichol, 2008; Treanor, 2004) antivirals directed against the virus neuraminidase have become an important addition to the armoury providing relief from influenza disease (Colman, 2009; Oxford, 2007; Smith et al., 2006). The antivirals oseltamivir and zanamivir protect against all influenza A and B strains (Jefferson et al., 2009). Oseltamivir, taken orally, and zanamivir, a nasal spray, are administered twice daily, and are most effective when taken before or soon after infection. However, viruses are well-known for acquiring resistance, and oseltamivir-resistant mutants, already wide-spread in seasonal pre-2009 pandemic H1N1 virus (Hauge et al., 2009; Meijer et al., 2009), are now appearing in the 2009 pandemic virus (Duan et al., 2010; Hamelin, 2010; Ujike et al., 2011). New broad-spectrum counter-measures, which do not result in virus resistance, are urgently required.

Oseltamivir was preclinically tested in ferrets and these animals are the preferred model for studying study new viruses and investigating oseltamivir-resistant strains (Boltz et al., 2008; Govorkova et al., 2006, 2011; Hamelin et al., 2010; Herlocher et al., 2004; Itoh et al., 2009; Mendel et al., 1998). Thus we have used this model to compare the protective abilities of cloned DI 244/PR8 and oseltamivir. Data presented here show that a single intranasal dose of 2 μg of DI RNA is overall more effective than 10 doses of 2.5 mg/kg bodyweight administered twice daily over five days (25 mg/kg in total) of oseltamivir at ameliorating the effects of pandemic influenza virus A/California/04/09 (H1N1).

## 2 Materials and methods

### 2.1 Ferret studies

Ferret work was conducted according to UK Home Office legislation and was approved by the local ethical committee. Thirty outbred male ferrets (*Mustela putorius furo*), 3–4 months of age, weighing 860–1367 g (mean 1082 g), were obtained from Highgate Farm, UK. They were seronegative for antibodies to A/Cal as determined by haemagglutination-inhibition. Ferrets were separated into 4 groups each comprising five animals: groups were treated intranasally with +300 μg active 244 DI virus and infected with A/Cal 2 h later, treated with oseltamivir by oral gavage (see below) and infected with A/Cal 2 h later, infected with A/Cal, and inoculated with saline. An identifier chip (idENTICHIP, Bio-Thermo) was inserted subcutaneously into the scruff of each animal. Ferrets receiving 244 DI virus (see below) were sedated by isoflurane inhalation before intranasal delivery of 500 μl (250 μl per nostril) of a single dose of 2 μg of 244 RNA in 300 μg of carrier virus. Ferrets receiving oseltamivir treatment were given 2.5 mg/kg bodyweight administered by oral gavage twice-daily every twelve hours (5 mg/kg bodyweight/day) over a period of five days as used by others (Govorkova et al., 2007; Hurt et al., 2010), which is comparable to an oral prophylactic human dose of 75 mg/kg bodyweight/day (Ward et al., 2005). Oseltamivir phosphate was acquired as oseltamivir powder (Roche) for oral suspension and was reconstituted with sterile water to a final concentration of 12 mg/ml. The volume of oseltamivir solution required for each ferret was calculated from the weight of each ferret recorded each morning on the day of administration. This oseltamivir dose and schedule protected ferrets from the highly virulent H5N1 virus (A/Vietnam/1023/04) when administered at 4 h after infection and then twice daily for 5 days (Govorkova et al., 2007). Two hours after intranasal administration of 244 RNA and the first oseltamivir dose, ferrets were sedated by intramuscular injection of ketamine (17.9 mg/kg) and xylazine (3.6 mg/kg) then inoculated intranasally with 500 μl (250 μl per nostril) of 100 TCID_50_ 2009 influenza virus A/California/04/09 (A/Cal; H1N1). Solutions were prepared on the day of challenge and the titre of the virus confirmed by infectivity assay. Control groups were infected with virus or inoculated with saline. Rectal temperatures were measured daily. Ferrets were monitored twice-daily post-challenge throughout the course of the study for clinical signs of influenza infection (lack of activity, sneezing, nasal discharge, lack of appetite, weight loss and pyrexia). Clinical signs were scored as follows: loss of activity scored 0 for normal activity levels, 1 for reduced activity, and 2 if inactive; nasal discharge scored 0 for no discharge and 1 for a discharge; sneezing scored 0 for no sneezing, and 1 for sneezing; appetite was scored 0 for no loss of appetite, and 1 for loss of appetite. Nasal washes were collected from each ferret following ketamine and xylazine sedation (as above) at days 1–6 and then at days 8, 10 12 and 14 post-challenge. For each nasal wash, 2 ml of PBS were instilled by small multiple volumes into each nasal cavity with expectorate collected into a beaker. The study was terminated at 14 days post-challenge.

### 2.2 Production of cloned 244 DI virus by reverse genetics

244 DI RNA was generated spontaneously during the transfection of 293T cells with plasmids to make infectious influenza A/PR/8/34 (Dimmock et al., 2008; Subbarao et al., 2003). The haemagglutinin (HA) protein of the original 244/PR8 virus had a preference for cell receptors comprising α2,3-linked sialyl receptor sequences, so we reconstructed 244 DI virus with the HA protein of a PR8 virus that binds to both α2,6- and α2,3-linked sialyl receptors (Meng et al., 2010), so that DI RNA would be delivered to cells bearing both types of receptor, and thus protect against infectious viruses which recognise either type of receptor as described previously (Meng et al., 2010). The resulting mixture of 244/PR8 DI virus and infectious helper A/PR8 virus was purified by pelleting through sucrose. Stocks were resuspended in PBS, standardized by haemagglutination titration, and stored in liquid nitrogen. All DI virus stocks were tested for their ability to protect mice as described previously (Dimmock et al., 2008) prior to their use in ferrets (data not shown). Before inoculation into animals, helper virus infectivity was eliminated with a short burst (50 s) of UV irradiation at 253.7 nm (0.64 mW/cm^2^). This is referred to as ‘active DI virus’. The UV inactivation target is viral RNA, and UV has little effect on the DI RNA because of its small target size, 395 nt compared with 13,600 nt for infectious virus. The absence of infectivity after UV-irradiation was checked by infectivity assay (see Section 2.4) and by intranasal inoculation into mice (Dimmock et al., 2008).

### 2.3 PCR assays

RNA was extracted from nasal washes with a QIAamp mini RNA kit (Qiagen), and quantitative real time PCR performed to quantitate virion-sense RNA using an ABI prism 7000. The following primers and probe were used: 244 1F (5′ CTCTTTGCCCAGAATGAGGAAT 3′), 244 1R (5′ CATAATCAAGAAGTACACATCAGGAAGAC 3′) and probe (5′ FAM-CCCTCAGTCTTCTCC 3′). Primers were synthesized by Invitrogen, and the probes by ABI. Reverse transcriptase reactions (10 μl) were performed using 6 μl extracted RNA, RevertAid reverse transcriptase and random hexamer (Fermentas) according to the manufacturer’s instructions. cDNA (1 μl) was used in 20 μl of PCR reaction. A virion-sense 244 RNA standard was made by subcloning PCR products of full length 244 RNA in pGEMT-easy vector (Promega). RNA was transcribed using the T7 polymerase (MEGAscript, Ambion), the mix was digested with DNase I, and RNA purified by electro-elution. After ethanol precipitation, RNA was resuspended into RNase-free water and quantitated on a Nanodrop 1000 (Thermoscientific, Wilmington, DE). Standard curves were generated by performing 10-fold serial dilutions of known RNA copy numbers with each dilution assayed in duplicate. The reaction was conducted at 50 °C for 2 min, 95 °C for 10 min, then 40 cycles of 94 °C for 15 s followed by 60 °C for 1 min.

### 2.4 Infectivity assay

Nasal washes from each ferret were titrated for A/Cal infectivity in a focus-forming assay using MDCK cells in 96-well plates in triplicate (Scott et al., 2011a). After infection cells were incubated at 33 °C for 24 h, fixed overnight at 4 °C with 1:1 methanol:acetone, and blocked with 5% w/v milk powder in PBS. Virus-positive cells were detected using a mouse monoclonal antibody that recognises the NP protein of influenza A viruses (9G8 Abcam), and a goat anti-mouse IgG-alkaline phosphatase conjugate (Sigma), both in buffered saline containing 0.1% v/v Tween, and finally incubated with an alkaline phosphatase substrate (NBT/BCIP in TMN buffer; Sigma). At least 50 stained cells (foci) at an appropriate dilution were counted in each of three wells and averaged to give a titre in focus-forming units (FFU) per ferret. Assays carried out on different days were normalised to a standard A/Cal virus preparation. Variation in the standard was less than 4-fold.

### 2.5 Haemagglutination-inhibition (HI) assay

Before assay sera were treated with receptor destroying enzyme (RDE II (SEIKEN), Cosmos Biological) overnight at 37 °C to remove non-specific inhibitors of haemagglutination and then incubated at 56 °C for 30 min to destroy the enzyme. Serial 2-fold dilutions of serum were incubated with 4 HAU of A/Cal for 1 h at ambient temperature before adding chicken red blood cells (VLA, Weybridge). The HI titre is expressed as the reciprocal of the dilution of serum that causes 50% inhibition of agglutination, and is interpolated between full agglutination and no agglutination.

## 3 Results

### 3.1 244 DI virus reduces weight more effectively than oseltamivir in A/Cal-infected ferrets

**Fig. 1:**
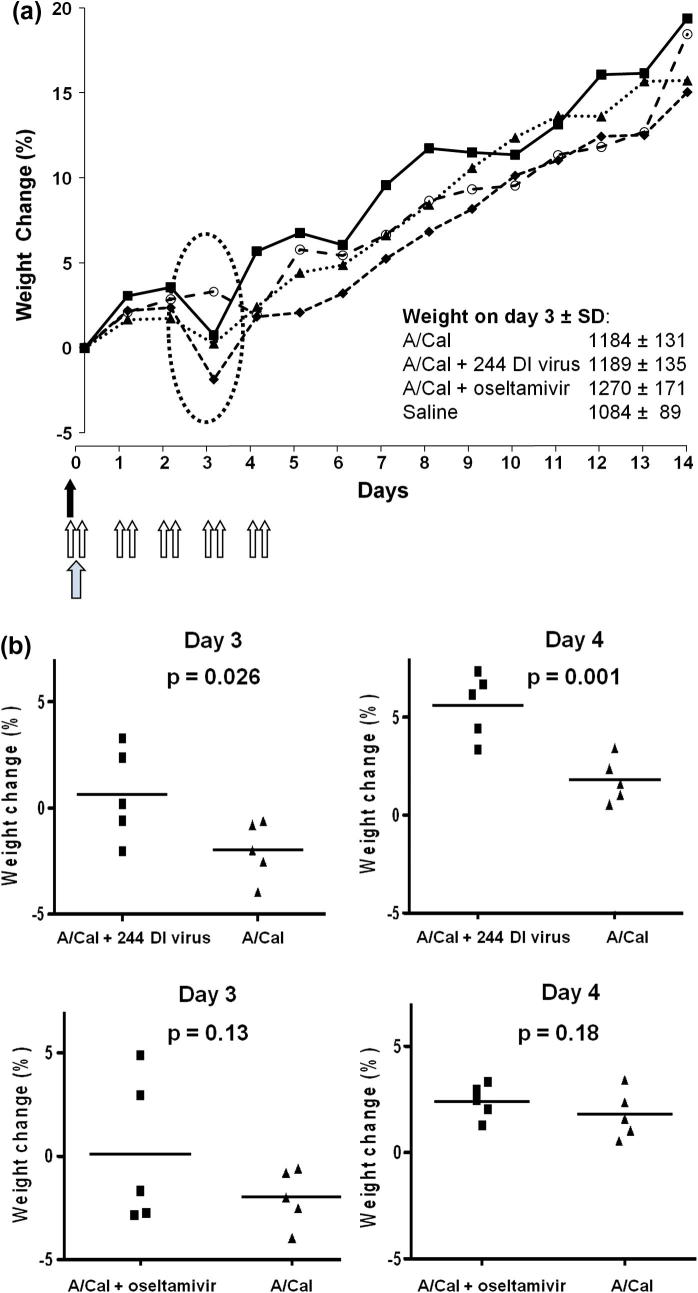
Changes in weight of A/Cal/04/09-infected ferrets given one dose of 244 DI virus at 2 h before infection (solid arrow) or 10 treatments with oseltamivir (open arrows) also given at 2 h prior to infection (stippled arrow) and then twice daily over 5 days. Ferrets (*n* = 5) were treated on day 0 and infected 2 h later. Each arrow represents one treatment. The data show the mean group body weight changes expressed as a percentage change compared to the group average weight at day 0 (a) The mean group weight loss in infected ferrets was reduced on day 3 by 244 DI virus and by oseltamivir (circled); means ± SD are inset. Only 244 DI virus had a significant effect (see panel b). ■, A/Cal + 244 DI virus; ▴, A/Cal + oseltamivir; ♦, A/Cal; ○, saline. Data from individual animals and the results of statistical analysis using a one tailed unpaired t-test for data from days 3 and 4 post infection is shown in panel (b). 244 DI virus-treated infected ferrets lost significantly less weight than untreated infected ferrets on days 3 and 4. The mean weight change of oseltamivir-treated, infected ferrets did not differ significantly from that of untreated infected ferrets on days 3 and 4.

Analyses of the weights of the animals and the percentage weight changes relative to the weight on day 0 were carried out with a repeated measures ANOVA. This was conducted on the data for days 1 to day 8 post-infection, as these reflect the period during which infectious virus was detected in nasal washes (see Section 3.5). Data confirm that infection significantly affected weight compared with non-infected animals throughout the 8 day period (*p* < 0.05). The data also show a single treatment of infected ferrets with 244 DI virus resulted in a greater overall weight gain that was seen with the infected control animals (*p* < 0.05). This indicates that while the treated animals experienced a transient weight loss on day 3 (Fig. 1a), this was less than was seen with the infected control group, and that treated animals subsequently gained weight at a greater rate than did the control infected animals. In contrast the repeated measures ANOVA showed that while multiple (10) treatments with oseltamivir resulted in a reduced weight loss when compared with the infected control group (Fig 1a), this was not significant at the 5% level. The repeated measures ANOVA identified day 3 post-infection as the time at which the greatest difference between either of the two treatments and the control infected group occurred, with DI virus providing the more effective amelioration of weight loss. Separate analysis of data using a one tailed unpaired t test was in agreement with the repeated measures ANOVA. The t-test showed that a single treatment with 244 DI virus at 2 h prior to infection significantly protected ferrets from A/Cal-associated weight loss on days 3 and 4 (Fig. 1b). By *t*-test oseltamivir, given at 2 h prior to infection and overall twice daily for 5 days, did not significantly reduce weight loss compared to the untreated infected group on days 3 and 4 (Fig. 1b).

### 3.2 244 DI virus and oseltamivir reduce fever in A/Cal-infected ferrets

**Fig. 2:**
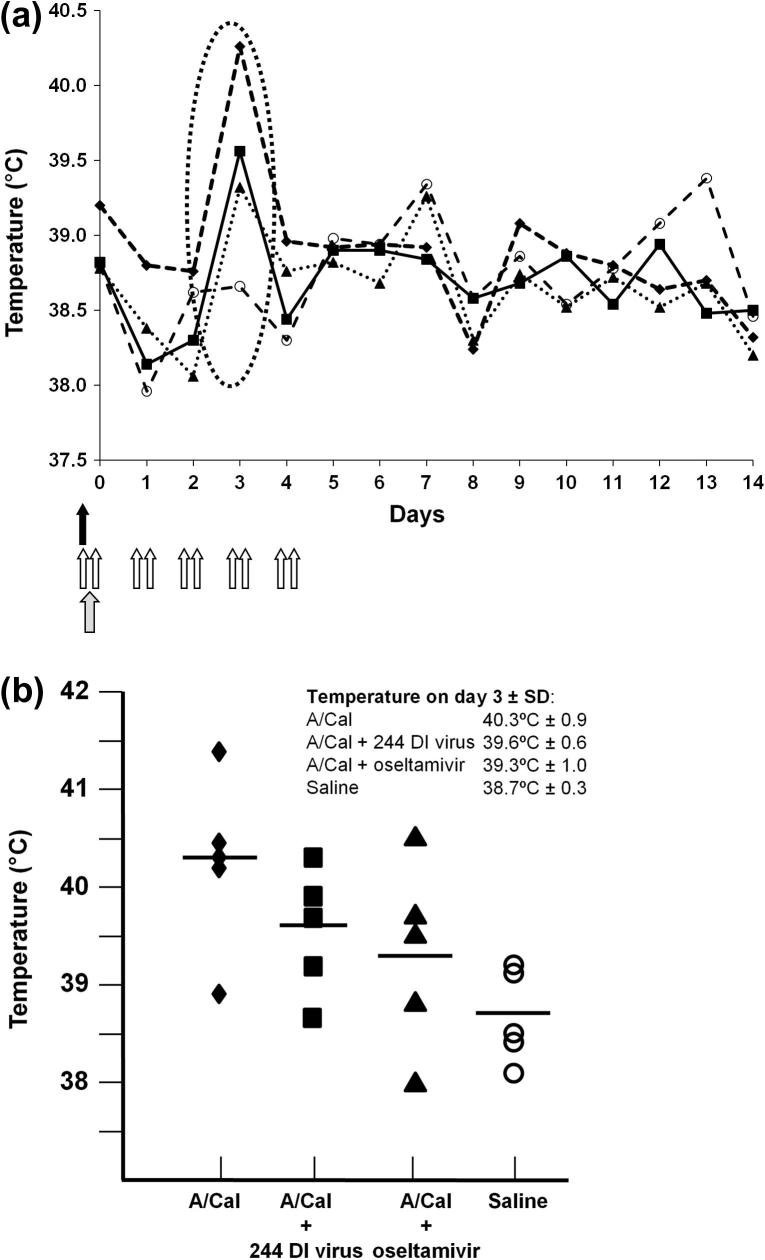
Temperatures in A/Cal-infected ferrets given a single treatment of 244 DI virus (solid arrow) or 10 treatments with oseltamivir (open arrows). (a) Temperatures over the period of the study. Infection is denoted by a stippled arrow. The key region of temperature elevation on day 3 is circled. (b) Details of the temperatures of individual animals on day 3 are shown with standard deviations. Other data are as in Fig. 1. Animals (*n* = 5) were anaesthetised and rectal temperatures taken prior to any other procedure. ■, A/Cal + 244 DI virus; ▴, A/Cal + oseltamivir; ♦, A/Cal; ○, saline.

Fig. 2a shows the mean daily temperatures for the groups of ferrets following infection. Control A/Cal-infected ferrets developed a pronounced fever spike at 3 days after infection (circled). Fever was reduced by both 244 DI virus and oseltamivir treatments. Though the reduction in temperature on day 3 post infection with either treatment was clearly evident, neither was statistically significant at the 95% level (*p* = 0.09 and *p* = 0.07 for treatment with 244 DI virus or oseltamivir treatment, respectively). This was due to one ferret in the control A/Cal-infected group that recorded a non-elevated temperature, as omission of this data point gave *p* values of 0.02 and 0.04 for treatment with 244 DI virus or oseltamivir, respectively (Fig. 2b). There was no statistical difference in the day 3 temperatures in infected ferrets treated with 244 DI virus or oseltamivir (*p* = 0.32).

### 3.3 244 DI virus but not oseltamivir reduces respiratory disease (sneezing and nasal discharge) in infected ferrets

**Fig. 3:**
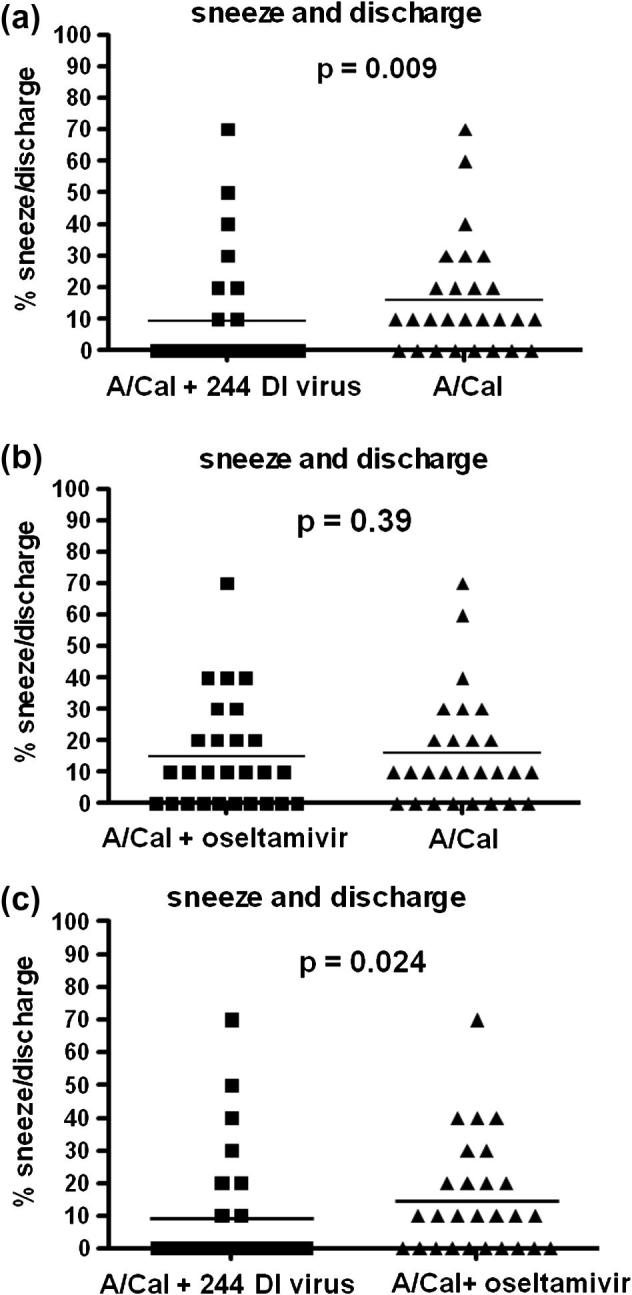
A single treatment with 244 DI virus, but not 10 treatments with oseltamivir, significantly reduced respiratory disease (sneezing and nasal discharge) in infected ferrets. Data collected over 14 days were analysed. The values are the occurrence of sneezing and nasal discharge events in each group over the 14 days of observation. The scores were assigned 10% for each individual event, so that if all five ferrets were positive for both sneezing and nasal discharge they would score 100%. Zero is only scored when the other group was positive. Statistical significance was determined with a one tailed Mann–Whitney U test.

Ferrets were monitored for sneezing and nasal discharge, both typical respiratory signs of influenza. Analysis of data collected twice daily (morning and afternoon) over the 14-day duration of the study showed that treatment with 244 DI virus significantly (*p* = 0.009) reduced the score compared with the infected A/Cal control group by 1.7-fold (Fig. 3). Oseltamivir treatment gave no significant reduction in respiratory disease (Fig. 3). Infected ferrets treated with 244 DI virus had significantly fewer combined clinical signs (1.6-fold) than those treated with oseltamivir. There was no difference in the time of respiratory disease between the 244 DI virus-treated group and the oseltamivir-treated group.

### 3.4 244 DI virus and oseltamivir reduce cellular infiltration into the nose of infected ferrets

The appearance of a cell infiltrate in nasal washes is a general response to respiratory infection in ferrets. On day 2 the influx of cells in control A/Cal-infected animals was significantly reduced 5-fold by treatment with 244 DI virus and 9.6-fold by oseltamivir (Table 1). On day 3 cell influx was again significantly reduced 1.8-fold by 244 DI virus and 10.7-fold by oseltamivir. However, despite the apparently higher reduction by oseltamivir, the outcome of the two treatments did not differ significantly (Table 1). By day 4 cell infiltration had increased in all groups to a similar level, approximately 100-fold above background. This remained at a plateau for around 8–10 days and then slowly decreased. Cell levels were still elevated by approximately 10-fold on day 14 when the study was terminated, although the level in the 244 DI virus-treated infected ferrets was 2.5-fold lower than in oseltamivir-treated infected animals (Table 1).

### 3.5 244 DI virus and oseltamivir reduce nasal wash infectivity in infected animals compared to control A/Cal-infected ferrets

**Fig. 4:**
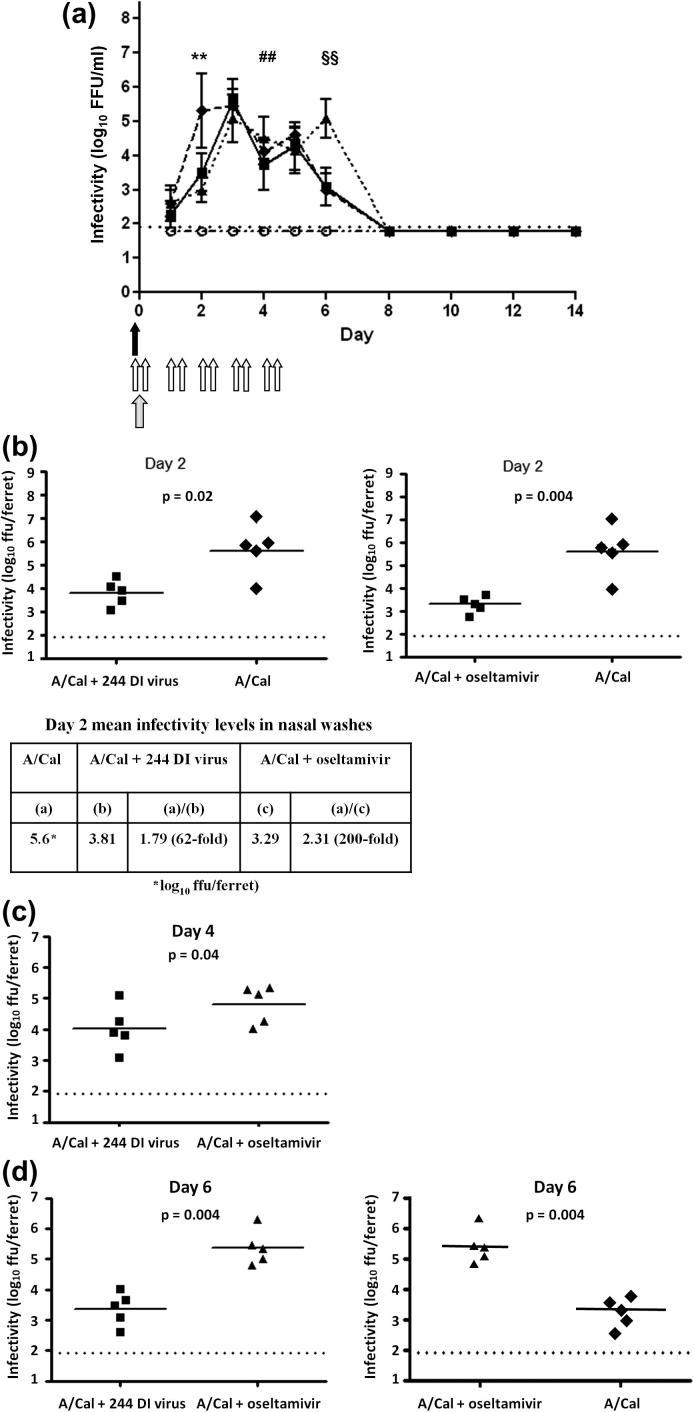
A/Cal infectivity in nasal washes of infected ferrets given a single treatment with 244 DI virus (solid arrow) or 10 treatments with oseltamivir (open arrows). Time of infection is denoted by the stippled arrow. Infectivity titres were titrated in MDCK cells (a). The limit of sensitivity of the assay was 1.92 log_10_ FFU/ml (dotted line). ■, A/Cal + 244 DI virus; ▴, A/Cal + oseltamivir; ♦, A/Cal; ○, saline. Panel b: on day 2, 244 DI virus and oseltamivir significantly reduced virus infectivity by 62-fold and 200-fold respectively (one-tailed Mann–Whitney U test) (^∗∗^in panel a). Panel c: on day 4 infectivity was significantly lower (5.9-fold) in 244 DI virus-treated ferrets (mean 4.03 log_10_) on day 4 than in oseltamivir-treated ferrets (mean 4.8 log_10_) (^##^ in panel a). Panel d (left): on day 6 infectivity was significantly higher (105-fold) in oseltamivir-treated infected ferrets (mean 5.38 log_10_) than in 244 DI virus-treated infected animals (3.37 log_10_), or 123-fold higher than in control infected animals (mean 3.29 log_10_) (panel d right, and ^§§^in panel a).

Infectious virus in the control A/Cal-infected group was just above background on day 1 after infection, and by day 2 had increased by more than 100-fold to 10^5.6^ ffu per ferret (Fig. 4a). The levels of infectious virus detected on day 2 in the 244 DI virus-treated, infected group was 62-fold lower, and the oseltamivir-treated group was 200-fold lower (Fig. 4b). The difference between infectivity titres in the 244 DI virus-treated and infected group and the oseltamivir-treated and infected group was not significant. On day 4 the infectivity titre in the 244 DI virus-treated infected group was 6-fold lower than in the oseltamivir-treated infected groups on day 4 (*p* = 0.04; Fig. 4c). Titres began to fall from day 4 and by day 6 those in the 244 DI virus-treated infected group and the untreated infected group had fallen to 10^3.4^ and 10^3.3^ ffu per ferret, respectively. However, on day 6 the infectivity of the oseltamivir-treated infected group was 123-fold higher than the control infected group (10^5.4^ ffu per ferret), a highly significant difference (*p* = 0.004; Fig. 4d). All five animals in the oseltamivir treated group had high titres of infectious influenza virus. The possibility that the influenza virus had developed resistance to oseltamivir was investigated by determining if the virus from the oseltamivir-treated infected group had developed the H275Y amino acid change that frequently accompanies resistance to oseltamivir. This was not found and the reason for high infectivity titres and/or slower virus clearance in the presence of oseltamivir is not known. Infectivity in all groups was undetectable by day 8, showing that 244 DI virus did not compromise virus clearance or lead to persistence of virus infectivity.

### 3.6 244 DI virus RNA in ferret nasal washes

**Fig. 5:**
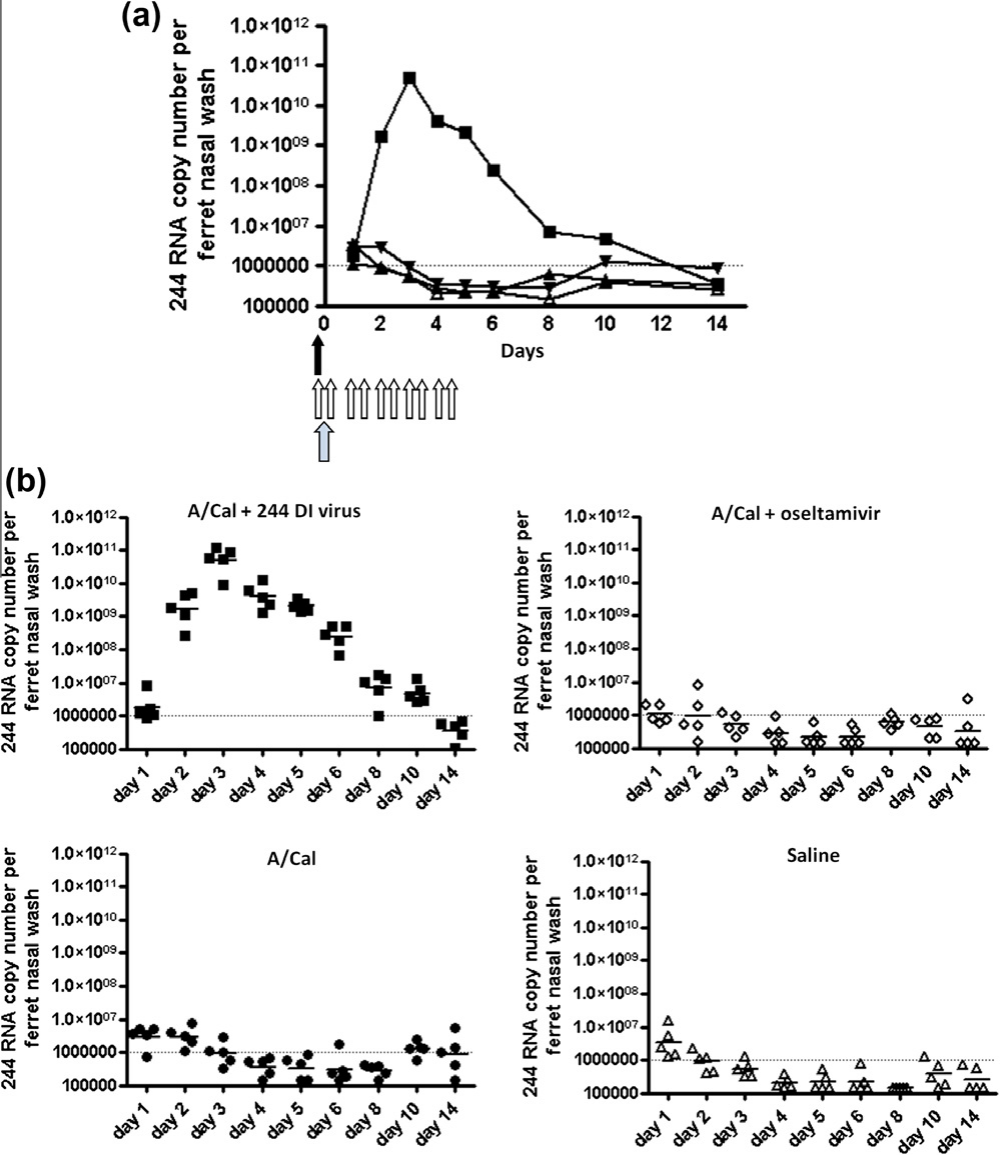
Amplification of 244 DI RNA in nasal washes taken from A/Cal-infected ferrets given a single treatment with 244 DI virus (solid arrow) or 10 treatments with oseltamivir (open arrows). Time of infection is denoted by the stippled arrow. The total amount of 244 DI RNA present in each nasal wash was determined by quantitative RT-PCR. The dotted line shows the limit of detection. (a) Mean 244 RNA copy numbers for each ferret group (*n* = 5). ■, A/Cal + 244 DI virus; ▴, A/Cal + oseltamivir; ▾, A/Cal; Δ, saline. (b) 244 DI RNA values for individual ferrets.

RNA was extracted from the nasal washes above and assayed by quantitative RT-PCR for the presence of 244 DI RNA, the active principle responsible for DI virus-mediated protection against disease caused by influenza A viruses (Dimmock et al., 2008). Fig. 5a summarizes the mean values per ferret group per day, and Fig. 5b show values for individual animals. The data show that 244 DI virus RNA was marginally above detectable levels at 1 day after infection, and that by day 2 there were high levels of 244 DI virus RNA in infected ferrets treated with 244 DI virus. 244 DI virus RNA was not detected in the other groups indicating that 244 DI virus RNA is specific for ferrets inoculated with 244 DI virus. The 244 DI virus RNA levels increased by nearly 1000-fold between days 1 and 2, and by over 25,000-fold between days 1 and 3, reaching a peak of 10^10.8^ copies per ferret. 244 DI virus RNA then steadily declined reaching a very low level on day 10 and was undetectable on day 14 after infection. These data demonstrate the ability of the 244 DI virus RNA to be amplified by the very agent that it is acting against – in this case A/Cal influenza virus. The >25,000-fold expansion factor effectively augments the applied dose of 244 DI virus RNA from 2 μg to >50 mg per animal.

### 3.7 Other clinical parameters

In addition to the signs and symptoms described above ferrets were monitored in the morning and the afternoon for loss of appetite, appearance of diarrhoea, and reduction in activity. None of these was seen in any group.

### 3.8 244 DI virus does not inhibit the development of HI serum antibody

There was a strong HI antibody response to A/Cal/04/09 (H1N1) in sera taken at 14 days after infection whether groups had been treated with 244 DI virus, oseltamivir or were untreated (Table 2). The titre of 244 DI-treated infected group was significantly higher than the infected group treated with oseltamivir (*p* = 0.008). Thus amelioration of infection by 244 DI virus appeared to improve rather than diminish the antibody response to the A/Cal haemagglutinin.

## 4 Discussion

In this study we compared the effects of treatment with DI virus and oseltamivir on the course of disease and virus load resulting from infection with 2009 pandemic influenza A virus (A/California/04/09) in the ferret. Data summarized in Table 3 show that DI virus reduced fever, weight loss, respiratory symptoms (sneezing and nasal discharge), the appearance of cells in nasal washes, and virus load. All this was coincident with a dramatic increase in DI RNA, presumably due to its amplification by A/Cal. It is reasonable to suppose that the beneficial effects are the result of the action of this augmented population of DI RNA. Augmentation of 244 DI RNA is consistent with the mouse model in which amplification of 244 DI virus RNA by various influenza A viruses has been documented many times (Dimmock et al., 2008; Scott et al., 2011a). It is likely that 244 RNA in nasal washes is packaged into newly synthesised DI virus particles as influenza RNA either free or in a ribonucleoprotein structure is susceptible to degradation by ribonuclease (Duesberg, 1969). Furthermore, new DI RNA is likely to be present as A/Cal DI particles as little, if any, of the A/PR8 genomic RNA survives the UV irradiation which is given specifically to destroy any infectivity present (see Methods). These A/Cal DI particles can transmit the antiviral 244 DI virus to other cells in the respiratory tract, and progressively increase the number of cells that are able to resist infection through the presence of DI RNA. Treatment with DI virus did not adversely affect the clearance of virus infectivity, and DI RNA was cleared from nasal secretions at a similar rate. The role of interferon in protection of ferrets from A/Cal was not investigated. Studies in mice showed that active DI virus given intranasally in the absence of infectious virus stimulates production of interferon type I in the lung, and that the UV-inactivated DI virus did not stimulate detectable interferon type I in the lung. However, use of interferon receptor knock-out mice showed that interferon was not required for protection against type A influenza virus (Dimmock et al., 2008), but did protect mice from pneumonia virus of mice and an influenza B virus (Easton et al., 2011; Scott et al., 2011b). UV-inactivated DI virus did not protect, and thus does not induce interferon type I.

Oseltamivir treatment was also beneficial although it did not cause any significant decrease in weight loss or respiratory disease when compared to the infected animals that were not given any other treatment. Oseltamivir reduced virus load on day 2, but the virus load in oseltamivir-treated animals was more than 100-fold greater than the virus control on day 6. This differential appears consistent with a viral rebound observed following the cessation of oseltamivir treatment in people infected with pandemic 2009 virus (Lee et al., 2011). We also examined virus from oseltamivir-treated ferrets on day 6 by sequencing for the H275Y mutation that is associated with resistance to oseltamivir (Ives et al., 2002) but this mutation was not found (unpublished data). The H275Y mutation was also absent from rebound virus in the oseltamivir-treated human cohort (Lee et al., 2011). We surmise that the rebound virus may result from the release of progeny virus that had before been bound to cell receptors because of the inhibition of viral neuraminidase activity by oseltamivir.

All ferrets developed A/Cal-specific, serum HI antibody, but there was significantly less in the oseltamivir-treated infected group than in the DI virus treated infected group. In addition to the signs and symptoms described above ferrets were monitored in the morning and the afternoon for loss of appetite, appearance of diarrhoea, and reduction in activity. None of these was seen in any group.

We conclude that treatment of ferrets with 244 DI virus ameliorates clinical disease and virus load resulting from infection with pandemic A/California/04/09 (H1N1) more effectively than did treatment with oseltamivir. DI virus did not cause virus to become persistent or delay clearance, and the presence of high levels of HI antibody in DI-treated, A/Cal-infected animals suggest that ferrets would be able to resist a repeat dose of virus.

## Figures and Tables

**Table 1:** Quantitation of cells in nasal washes: 244 DI virus and oseltamivir reduced the number of cells in nasal washes in A/Cal infected ferrets on days 2 and 3.^a^

Group	Days relative to infection on day 0														
	−4	1	2	3	4	5	6	7	8	9	10	11	12	13	14
A/Cal	4.7 ± 0.1	5.0 ± 0.2	6.0 ± 0.2	7.4 ± 0.3	7.0 ± 0.2	7.0 ± 0.1	7.2 ± 0.1	∗	6.8 ± 0.2	∗	6.7 ± 0.2	∗	6.5 ± 0.2	∗	6.0 ± 0.5
244 DI virus + A/Cal	5.0 ± 0.2	4.8 ± 0.2	**5.3** ± **0.2**	**7.1** ± **0.3**	6.9 ± 0.4	6.9 ± 0.4	7.2 ± 0.2	∗	6.6 ± 0.3	∗	6.7 ± 0.2	∗	6.4 ± 0.3	∗	5.7 ± 0.5
Oseltamivir + A/Cal	4.7 ± 0.2	5.0 ± 0.2	**5.0** ± **0.3**	**6.3** ± **0.9**	7.1 ± 0.4	7.1 ± 0.3	7.2 ± 0.1	∗	7.1 ± 0.2	∗	6.9 ± 0.3	∗	6.8 ± 0.1	∗	6.1 ± 0.3
Saline	5.2 ± 0.3	4.9 ± 0.4	5.1 ± 0.2	4.9 ± 0.2	4.9 ± 0.2	4.8 ± 0.2	4.8 ± 0.1	∗	4.8 ± 0.2	∗	4.8 ± 0.2	∗	4.8 ± 0.3	∗	4.9 ± 0.1

a Mean log_10_ cells per ferret ± SD; ⁎a nasal wash was not carried out on this day; on days 2 and 3 the 244 DI virus- and oseltamivir-treated groups (bold) were significantly different from the A/Cal group but not from each other.

**Table 2:** HI serum antibody determined before infection and at 14 days after infection.^a^

Treatment and inoculum	HI titre on			
	Day 0	Day 14		
244 DI virus + A/Cal	<47	8280 ± 2696		
			*p* = 0.008	
Oseltamivir + A/Cal	<47	4533 ± 593		*p* = 0.22
			*p* = 0.095	
A/Cal	<47	6666 ± 3772		
Saline	<40	<47		

a The HI titre (± SD) is the reciprocal of the serum dilution giving 50% inhibition of haemagglutination. Significance was determined using a two tailed Mann–Whitney U test. The HI titres of ferrets given 244 DI virus +A/Cal and A/Cal alone were not significantly different on day 14 (*p* = 0.22).

**Table 3:** Summary of protection of ferrets from A/Cal-mediated infection and disease using 244 DI virus or oseltamivir.

Comparison	244 DI virus	Statistically significant	Oseltamivir	Statistically significant	Data source
Reduction in loss of body weight compared with the mock-treated infected group	Yes	Yes	No	No	Fig. 1
Subsequent weight gain compared with the mock-treated infected group	Yes	Yes	No	No	Fig. 1
Reduction in fever compared with the mock-treated infected group	Yes	No	Yes	No	Fig. 2
Reduction in combined sneezing & nasal discharge compared with the mock-treated infected group	Yes	Yes	No	No	Fig. 3
Reduction in cells in nasal wash compared with the mock-treated infected group	Yes	Yes	Yes	Yes	Table 1
Reduction of infectivity in nasal washes on day 2 compared with the mock-treated infected group	Yes (and on day 4)	Yes	Yes	Yes	Fig. 4
Reduction of infectivity in nasal washes on day 4 compared with the oseltamivir-treated infected group	Yes	Yes	No	No	Fig. 4
Reduction of infectivity in nasal washes on day 6 compared with the oseltamivir-treated infected group	Yes	Yes	No: higher than the virus control	N/a	Fig. 4
Enhancement of 244 DI RNA in nasal washes	From day 2; maximum mean increase 25,000-fold	Yes	244 RNA absent	N/a	Fig. 5
Development of A/Cal-specific HI antibody	Yes	Yes	Yes	Yes	Table 2
